# Prolonged apnea after ECT in organophosphorus poisoning – the need to redefine norms

**DOI:** 10.1186/s12888-021-03150-0

**Published:** 2021-03-10

**Authors:** Shibu Sasidharan, Harpreet Singh Dhillon

**Affiliations:** 1 HOD, Department of Anaesthesiology & Critical Care, 150 GH, Rajouri, Jammu & Kashmir, India; 2 Department of Psychiatry, 166 Military Hospital, Jammu, India

**Keywords:** Organophosphorus poisoning, Succinylcholine, Prolonged apnea, Pseudocholinesterase, Depression, Electroconvulsive therapy, MECT

## Abstract

**Background:**

Poisoning and deaths by organo-phosphorous (OP) compounds are one of the major causes of death in developing and poor countries, and a common admission in the emergency ward and the ICU. OP compounds act by irreversibly binding to pseudocholinesterase enzyme and hence prolong the apnea in patients being given suxamethonium. We present a unusual case of OP poisoning (OPP) in which prolonged apnea ensued in a patient of severe depression following MECT (modified electroconvulsive therapy) in which suxamethonium was used as muscle relaxant, in whom we were cautious of the side-effect of prior organophosphorus poisoning. Since the cases of OPP are very high worldwide, a thorough knowledge of the interaction of the action of the drug and the receptors on which it acts takes pride of place. This article highlights the nuances in the field of psychiatry and anaesthesia in diagnosis and management of prolonged apnea after ECT.

**Case presentation:**

A 53/F patient consumed OP 38 days prior to MECT. Since existing literature recommend a delay of 4 weeks and a subminimal dose of suxamethonium to prevent prolonged apnea, both these points were taken into consideration. Despite 38 days post exposure to OP, and a dose of succinylcholine of < 0.3 mg/kg, the patient remained apneic for 3 h. Suxamethionum apnea was managed with elective ventilation. After recovery, patient had no residual effect. Subsequently her pseudocholinesterase levels were done which were found to be very low.

**Conclusion:**

This case is being presented to emphasize that behaviour of post synaptic receptors cannot be relied upon after OP poisoning and pseudocholinesterase levels needs to be mandatorily checked, irrespective of duration post-exposure. In strong suspects dibucaine number and fluoride number also needs to be estimated.

## Background

A 53 year-old lady presented to a tertiary level military hospital in India, with alleged history of suicidal attempt by organophosphate pesticide poisoning (OPP). After treating the poisoning, she was referred to the psychiatrist for her probable mental illness. She was advised MECT, which was done using succinylcholine as muscle relaxant. After administration of succinylcholine, she required prolonged ventilatory support of 3 h, subsequent to delayed recovery from the drug. We present this case to emphasize the fact that despite knowing her OPP status and its effect of anaesthesia, and taking all adequate precautions as per existing literature, the elimination of suxamethonium was not as expected, which necessitates the need to do a cholinesterase estimation in all cases of known/suspect OPP before suxamethonium use, irrespective of duration since poisoning.

## Case presentation

A 53-year old married female, known case of Depressive disorder for the last 02 years on medication with poor compliance presented to our hospital with an alleged history of attempted suicide by ingesting Organo-phosphorous (OP) compound (composition not known) 02 months back. The patient was managed for OP poisoning in ICU following which a psychiatric consult was sought. She gave a month’s history of persistent sadness of mood; loss of interest in previously pleasurable activities, easy fatigability; significantly reduced sleep, appetite, and minimal to none social interactions since around 03 months. She would at times exhibit bouts of anger outbursts without any apparent cause, followed by periods of extreme hypo-activity manifested by sitting alone in a particular posture. Gradually, the symptoms worsened and 5–6 days before she was brought to the hospital, she had become almost mute and would remain confined to her bed. It was observed by her family members that she was mumbling to the self along with hand gesticulations in the air and had to be consistently coaxed for eating/elimination needs. Patient firmly believed that people around her including family members were talking ill about her and were planning to somehow get rid of her. On further probing, patient justified her beliefs by considering herself as a burden on her family members in view of her long-standing non-remitting illness. She also acknowledged thoughts of self-harm as her last resort to put an end to her suffering. Then, on the fateful day, the patient consumed an unquantified volume of rat poison kept in the storehouse with an intent to die. She was found lying on the floor by family members smelling of garlic odour and was then brought to hospital. She gave past history of fracture left forearm with uneventful complete recovery (open reduction and internal fixation under GA) 16 years back. There was no family history of psychiatric illnesses. Personal history was characterized by anxious-avoidant traits with low stress tolerance. There was no history of any substance use in dependent/abuse pattern. On examination, her pulse was 86/min and blood pressure (BP) was 126/88 mmHg. She was ill kempt, had severe psychomotor retardation, mutism, depressed mood with flat affect, depressive cognitions of helplessness and hopelessness along with mood congruent delusions of reference and persecution. She denied any perceptual abnormalities in clear sensorium and had poor insight with deranged biorhythms. Relevant investigations including NCCT head, renal function tests, serum electrolytes, random blood glucose levels, electrocardiogram, liver function tests, and thyroid profile were within normal limits. An impression of a severe depressive episode with psychotic symptoms was made (ICD 10 F32.3). Patients and family members were counseled regarding treatment options and ECT was offered as the first choice, which was declined by patient as well as family members. She was then started on Cap Fluoxetine 20 mg OD along with Tab Olanzapine 5 mg HS and Tab clonazepam 01 mg HS. Her family members were counseled to be on vigilant round the clock monitoring, ensure patients compliance for medication, and a weekly review in the OPD. Patient however, did not show any significant improvement over the next 1 month despite gradually uptitrating her antidepressants and intensive Cognitive behaviour therapy (CBT) sessions. The patient was still having persistent sadness of mood with severe psychomotor retardation, active suicidal intent, and significantly impaired sleep and appetite. She was hence, again offered MECT as a treatment option, which was agreed upon by patient as well as family members.

The patient was now slated for MECT under general anaesthesia (GA). Pre-anaesthesia checkup (PAC) was done, in which a detailed history of the medications taken and past history was taken. Her son gave a history of OP poisoning a month back (38 days), and it was endorsed in the record. With proper advice, she was accepted in ASA Physical Status II (for mental illness on medication) for MECT under GA.

The patient was taken up for MECT. The patient had stable hemodynamics. Patient weighed 62 kgs, and was premedicated with Inj Glycopyrolate 0.2 mg, Inj Midazolam 1 mg, Inj Propofol 70 mg and a subminimal dose of Inj Scolene (15 mg, ie < 0.3 mg/kg) due to the prior history of OP poisoning.

MECT was given by the psychiatrist after complete muscle paralysis with a current constant machine. After GTCS, the patient was oxygenated by bag and mask ventilation. However, the patient did not resume spontaneous breathing even after 10 min. The expected time to return of spontaneous breathing after a full dose of succinylcholine is 9–11 min [1]. Despite the minimal dose of succinylcholine that was administered in this case, and having an adequate time gap since the episode of poisoning (38 days), the patient regained spontaneous breathing only after 3 h. Hemodynamics were stable throughout. Her ABG was done to assess status and all values were within normal limits. On the day of the procedure, there was no ventilator readily available in the ICU to handle this contingency, since such a complication is not anticipated routinely. Hence, the patient was ventilated using an i-gel and bag ventilation with occasional boluses (10 mg) of Inj Propofol. After the patient was fully conscious and regained full sensory and motor function, she was kept admitted for 24 h under observation in the HDU. She was asymptomatic throughout and was discharged the next day. Her psychotropic medications (as mentioned earlier) were continued, excluding Clonazepam. The subsequent 06 MECTs (every third day) were administered using alternative muscle relaxants pancuronium following which she had significant improvement in her mood symptoms without any side-effects/complications. She was then continued on medications along with CBT and was advised weekly OPD reviews with the psychiatrist.

To rule out any other cause and confirm diagnosis of suxamethonium apnea, her serum butyrylcholinesterase were checked, and the levels were as low as 240 u/l (normal reference range is 5900 and 13,200 u/l) [2]. Dibucaine and fluoride numbers were not analysed (due to lack of testing facility). Her family was also investigated to rule out genetic involvement, and their levels were within normal limits. Patient’s serum salicylate levels were negative, and RFTs were normal.

## Discussion & conclusions

It is difficult to forecast the behaviour of post synaptic receptors after OPP.

The normal onset & duration of paralysis following use of succinylcholine is 60 s and 8–10 min, respectively [1]. Prolonged paralysis after succinylcholine occurs if:

1. Patient has a genetic plasma cholinesterase functional abnormality, or deficiency, as is prevalent in some regions that have a high incidence of the atypical gene, including Egypt, north-west India and Turkey [3]. (Our patient had undergone surgery under GA for fracture forearm roughly a decade back and it was uneventful.)
2. Acquired decrease in plasma cholinesterase activity. (eg: Malnutrition, pregnancy and postpartum period, burns, liver disease, kidney disease, hemodialysis, MI, CHF, malignancy, chronic infections, and drugs such as steroids and cytotoxic agents can decrease the production of the pseudo cholinesterase enzyme [4]. All the above conditions were excluded in our patient.

Qualitative and quantitative defects of pseudocholinesterase activity have to be considered. The former are due to a genetic abnormality which results in the production of an enzyme inefficient in hydrolysing suxamethonium. Individuals at risk cannot be recognised by estimations of enzyme levels alone: dibucaine and fluoride numbers need to be determined. Simple depression of the normal enzyme may be associated with certain compounds used therapeutically (eg: cyclophosphamide, Ecothiophate iodide). Prolonged apnoea after suxamethonium is associated with the genetic group rather than with the quantitative reduction of the typical enzyme.

Organophosphate poisoning can produce irreversible inhibition of the activity of circulating plasma cholinesterase and result in prolonged respiratory paralysis [5]. Both suxamethonium and the non-depolarising muscle relaxant mivacurium are metabolised by plasma cholinesterases; therefore, their metabolism is reduced in the presence of OPP, resulting in a hyperbolic paralysis. The inhibition of acetylcholinesterase allows acetylcholine to accumulate at peripheral and central nervous cholinergic sites, causing prolonged paralysis. This phenomenon has been reasonably reported in adults and children with OPP and suxamethonium use. However, the duration of such an activity has been described as 04 weeks [6]. The gap between exposure to OPP and MECT was carefully considered in this case (38 days).

The ED95 of succinylcholine is less than 0.30 mg/kg [7] & doses as small as 0.40 mg/kg also provide clinically acceptable conditions for intubation. A study by Naguib et al. found that 0.60 mg/kg succinylcholine was sufficient to achieve acceptable intubating conditions at 60 s in 95% of patients anesthetized with 2 mg/kg fentanyl and 2 mg/kg propofol. Even doses as low as 0.30 mg/kg produced good or excellent conditions for intubation in 92% of patients. In this patient, a dose of < 0.3 mg/kg was given.

Non-depolarizing muscle relaxants like Inj. Vecuronium may produce severe bradycardia so Inj. Pancuronium remains the drug of choice. Requirement of muscle relaxant seems to be more due to high concentration of acetylcholine present in patient.

Inhalational agent like halothane should be used extremely carefully as chances of brady-arrhythmias are very high.

It is also suggested that medication combined with repetitive trans-cranial magnetic stimulation (rTMS) could be considered in the treatment of refractory depression, if available.

Despite knowing the history of OP poisoning and its effects on anaesthesia, the exaggerated response could not be prevented in this case (sub-minimal dose given and procedure after 38 days of injection).

This case brings to the fore on how it is mandatory to screen for OP poisoning prior to any MECT using succinylcholine. There are case reports on how this information has been concealed many times by the patient and has caused delayed recovery [8–10]. Serum cholinesterase estimation is not a routinely done investigation for OPP during PAC in India. If available dibucaine and fluoride numbers also need to be determined (which could not be on our patient due to unavailability).

Summarising, whenever a case of OP poisoning is being taken up for MECT, please consider:

1. Detailed history taking including medication history should be taken.
2. For any patient slated for MECT, in which succinylcholine is planned to be used, it is recommended that they be screened for OP Poisoning, irrespective of history. Screening for pseudocholinesterase deficiency [11] is routinely done in many centres by means of doing liver function tests and eliciting history if the patient belongs to the Vysya community [12], but pseudocholinesterase levels need to be checked specifically.
3. In strong suspects, dibucaine and fluoride numbers also need to be determined.
4. Non-depolarising agents (Vecuronium, Cis-atracurium, Rocuronium) can be considered for use, where muscle relaxation is required. In all such cases, ventilator backup should be available in view of prolonged muscle relaxation [12]. For every case of MECT, irrespective of history, optimum resuscitation efforts, including ventilator backup with adequate oxygen is essential.
5. The Role of Ketamine and Dexmeditomidine is extensively being studied as an effective alternative to MECT. This could be particularly advantageous in a resource scare country like India, where OP poisoning is common due to easy availability, the history of the same is often concealed, and sophisticated lab testing of relevant biomarkers (pseudocholinesterase) is limited [8].
6. *Suxamethonium Apnea* – Management:

The general principles of treatment that are well recognised include:

- Ventilation to maintain a normal Po2 & Pco2.
- Administration of double strength plasma [13]. Double strength plasma allows quantitatively supplementary enzyme to be infused without contributing to water load [14].
- The use of anticholinesterases.

## Data Availability

Can be requested from the author SS: shibusasi@gmail.com

## Abbreviations

OPP: Organo phosphorus poisoning
MECT: Modified electro-convulsive therapy
GA: General anaesthesia
BP: Blood pressure
NCCT: Non-Contrast Head CT
ICD: International Statistical Classification Of Disease & Related Health Problems
OD: Once daily
BD: Bi - daily
OPD: Out Patient Department
CBT: Cognitive behaviour therapy
PAC: Pre-anaesthesia checkup
GTCS: Generalised tonic clonic seizure
ABG: Arterial blood gas
HDU: High dependancy unit
RFT: Renal function test
